# Mobilizing political support proved critical to a successful switch from tOPV to bOPV in Kano, Nigeria 2016

**DOI:** 10.1186/s12889-018-6195-x

**Published:** 2018-12-13

**Authors:** Bashir Abba, Sule Abdullahi, Samuel Bawa, Kabir Ibrahim Getso, Imam Wada Bello, Charles Korir, Audu Musa, Fiona Braka, Adamu Ningi, Peter Nsubuga, Richard Banda, Sisay G. Tegegne, Faisal Shuaib, Usman Said Adamu, Sulaiman Haladu

**Affiliations:** 1 World Health Organization, Country Representative Office, Abuja, Nigeria; 2 Global Public Health Solutions, Atlanta, GA USA; 3 Ministry of Health, Kano, Kano State Nigeria; 4 National Primary Health Care Development Agency, Abuja, Nigeria; 5 Africa Field Epidemiology Network, Hospital Road, Kano, Kano State Nigeria

**Keywords:** Mobilization, Stakeholder, tOPV-bOPV switch, Circulating vaccine-derived poliovirus, Kano

## Abstract

**Background:**

Kano is one of the high-risk states for polio transmission in Northern Nigeria. The state reported more cases of wild polioviruses (WPVs) than any other state in the country. The Nigeria Demographic and Health Survey of 2013 indicated that OPV3 coverage in the routine immunization (RI) programmewas 57.9%. Additionally, serial polio seroprevalence studies conducted from 2011 to 2015 in the eightmetropolitan LGAs indicated low immunity levels against all three polio serotypes in children below one year. Areas with sub-optimal RI coverage such as Kanothat fail to remove all tOPV during the tOPV-bOPV switchwill be at increased risk of VDPV2 circulation.

**Methods:**

We assessed the impact of political leadership engagement in mobilizing other stakeholders on the outcomes of the bOPV-tOPV switch in Kano State from February to May 2016 using nationally-selected planning and outcome indicators.

**Results:**

A total of 670 health facilities that provide RI services were assessed during the pre-switch activities. Health workers were aware of the switch exercise in 520 (95.1%) of the public health facilities assessed. It was found that health workers knew what to do should tOPV be found in any of the 521 (95.2%)public health facilities assessed. However, there was a wide disparity between the public and private health practitioners’ knowledge on basic concepts of the switch.

There was 100% withdrawal of tOPV from the state and the seven zonal cold stores. Unmarked tOPVwas found in the cold chain system in 2 (4.5%) LGAs. Only one health facility (0.8%) had tOPV in the cold chain. No tOPVwas identified outside the cold chain without the “Do not use” sticker in any of the health facilities.

**Conclusion:**

The engagement of the political leadership to mobilize other key stakeholders facilitated successful implementation of the tOPV-bOPVswitch exercise and provided opportunity to strengthen partnerships with the private health sector in Kano State.

## Background

Poliomyelitis is an acute paralytic disease caused by three polioviruses (PV) serotypes. Less than 1% of PV infections result in acute flaccid paralysis [1]. The Sabin oral polio vaccine (OPV) and the inactivated polio vaccine (IPV) are used for active immunization against the disease. In 1988, when > 350,000 persons in 125 countries were affected by polio, the 41st World Health Assembly resolved to eradicate the disease by the year 2000 [2].

While OPV was instrumental in the success recorded in Polio Eradication Initiative (PEI), OPV has the disadvantage of genetic instability, resulting in cases of vaccine-associated paralytic poliomyelitis (VAPP) and vaccine-derived polioviruses(VDPVs) [3]. The potential risk of circulating vaccine-derived poliovirus (cVDPV) emergence has increased in recent years as wild poliovirus circulation has ceased in most of the world. The risk appears highest for the type 2 OPV strain because of its greater tendency to spread to contacts [4].

In 2013, the World Health Assembly endorsed a plan that calls for the ultimate withdrawal of OPV from all immunization programs globally. The withdrawal was to be conducted in a phased manner with the removal of the type 2 component of OPV in 2016 through a global switch from trivalent OPV to bivalent OPV [5]. IPV is introduced into routine immunization (RI) schedules in all countries to reduce the risk for cVDPV2 outbreaks and to facilitate responses to outbreaks. By the end of September 2015, 90 (46%) of 194 World Health Organization (WHO) member states were using IPV [6].

Nigeria made significant progress in polio eradication in recent years with the removal of the country from the list of endemic countries in September, 2015 by WHO. In April 2016, Nigeria participated in the switch exercise wheretOPVwas replaced with bOPV, the second objective of the Polio Eradication and Endgame Strategic Plan, 2013–2018 [7, 8].

Kano is one of the key high-risk states for polio transmission in Northern Nigeria. The state is also the most populous in the country. The state reported more cases of wild polioviruses (WPVs) than any other in the country. Kano has made significant progress in polio supplemental immunization activities (SIA) over the last few years. The proportion of Local Government Areas (LGAs) that attained coverage of 80% and above (assessed bylot quality assurance sampling (LQAS) surveys) rose from 60% in 2013 to 97% in 2016. However, this progress is precarious, because the Nigeria Demographic and Health Survey of 2013 indicated that OPV3 coverage in the routine immunization (RI) programme was 57.9% [9]. Also, serial polio seroprevalence studies conducted from 2011 to 2015 in the eightmetropolitan LGAs indicated low immunity levels against all three polio serotypes in children below 1 year [10]. Strong RI is essential for immunity against PV2 after the switch since IPV, delivered by RI program, would be the only source of PV2 antigen. Areas with sub-optimal RI coverage such as Kano will, therefore, be at increased risk of VDPV circulation. Accordingly every efforts should be made to remove all tOPV from all facilities to minimize this risk.

The interplay between politics and immunization in Kano is known to play a role in suspension of Polio Vaccination [11]. Misunderstandings and inadequate communication led Kano state to boycott polio immunization in 2003–2004 with devastating consequences to the global PEI [12]. The issue of vaccine derived polio virus was carefully handled and communicated to the public in the state. Immunization sceptics propagated the rumour that OPV was unsafe [13]. Therefore, any misinformation on the withdrawal of tOPV to prevent VDPV could play into the hands of these sceptics. Kano state health authorities felt there was a need for transparency, but the information should be passed in such a way that it contains no unnecessary details to spark controversy [14]. Additionally, there was a wide range of stakeholders handling tOPV in the state. Major stakeholders were private medical practitioners, pharmaceutical companies,and security agencies. Political leadership was required to effectively engage them for assured and complete withdrawal of tOPV. Thus, this paper describes the process of political leadership engagement of various stakeholders, the role played by individual stakeholders and the impact of this collaboration in the successful implementation of the switch between February and April 2016 in Kano.

## Methods

We conducted a cross-sectional descriptive study in Kano state, northern Nigeria from February to May 2016.

In doing so we targeted the leadership of Kano State Ministry of Health, the National Agency for Food and Drug Administration and Control (NAFDAC) and health professional associations that have representation in the state, public and private health facilities that provide RI service.

The National bOPV-tOPVSwitch Committee recommended the establishment of state switch committees to coordinate planning and implementation of the switch plan. The committee in Kano was headed by the Incident Manager (IM) at the State Emergency Operations Center (EOC). Members included state team leads of partner agencies:WHO, United Nations Children’s Fund (UNICEF), US Centers for Disease Control (CDC), Rotary International, Core-Group, and European Union Support to immunization Governance in Nigeria (EU-SIGN). The state switch committee established the state switch support team which comprised representatives of state primary health care development board (SPHCMB) and all partner agencies. The support team developed a detailed switch plan for the state and supported its implementation. Similar support teams were established in all the 44 LGAs.

The state switch committee paid an advocacy visit to the state Health Commissioner and briefed him on the switch plans and solicited for his active participation in the switch process. He was specifically requested to lead in the media engagement and other stakeholder engagement. The Health Commissioner convened series of meetings with representatives of private health practitioners where he highlighted the importance of the switch process and specific actions required from them. These included granting access to supervisors, allowing the withdrawal of any tOPV that may have been found and operating beyond their normal working hours if required. The Health Commissioner also issued a directive to all public health facilities in the state to ensure availability of RI focal persons even outside working hours in the week of the switch. Additionally, letters on the switch were written to commandants of all military and paramilitary health institutions in the state as well as weekly review meetings held on the challenges and the solution to the switch process under the Health Commissioner.

We informed key stakeholders about the switch process. The stakeholders included local government area (LGA) primary health care coordinators, medical directors of state general hospitals, private medical practitioners and the pharmaceutical society of Nigeria. NAFDAC which regulates pharmaceutical products was requested to be part of the supervision team to pharmaceutical companies and drug dealers that stock tOPV.

We trained all the personnel who participated in the switch data collection process. Data collection tools were developed at the national EOC. State and LGA level trainings were synchronized with SIA training for March 2016. On the job training for health facility staff were conducted during supervision by state supervisors. However, the training for independent switch validators was conducted by the national EOC. The switch had three stages: (1) pre-switch inventory of tOPV stock, (2) tOPV withdrawal and replacement with bOPV and (3) post switch validation. The pre-switch inventory stage involved identification and documentation of all tOPV stock, assessment of personnel’s knowledge on the switch and cold chain facilities. State pre-switch supervisors were identified from government and partner agencies.

Questionnaires were applied at LGA stores, satellite coldstores, and health facilities. Immediate feedback on the outcome of the supervision was provided at all levels. Weekly review meetings at state and LGA levels were conducted to discuss challenges and identify solutions.

The withdrawal stage involved removal of tOPV stock within and outside the cold chain. The withdrawn tOPV were marked “Do Not Use.” The Health Commissioner held a media briefing on the day of the switch to intimate the public on the switch and its objectives. The main purpose of the briefing was to pre-empt any mischievous misrepresentation on the dangers of tOPV that may be attempted by immunization skeptics. The validation stage involved inspection of all state, zonal and LGA cold stores for the presence of tOPV and bOPV. Similarly, health worker knowledge on the switch was assessed. The post switch validation was conducted from 19th to 21st April 2016 by independent monitors who were recruited outside the immunization system.

Data from the advocacy activities were summarized in MS word, while data on pre-switch supervision and post switch validation were collated and processed using MS Excel software package.

## Results

The Kano state Ministry of Health provided a clear leadership in the switch process. The Health Commissioner led the media engagement which was considered critical taking into account the sensitivity and the misunderstanding that often surround PEI interventions by the media in the state. Part of media engagement activities conducted by the commissioner was a media briefing on the switch day which attracted public and private media representatives. The commissioner highlighted objectives of the switch and the participants were given anopportunity to ask questions and seek clarifications on any issue about the switch.

NAFDAC provided valuable support during the planning and implementation of the switch. The agency provided information on pharmaceutical companies and other drug dealers that handled tOPV. Their staff also participated in the supervision of the pharmaceutical companies during pre-switch preparation which facilitated access to the companies (Table 1). The agency also supervised the destruction of 27,351 retrieved tOPV vials by the boil-and-bury method as outlined in the National Switch Committee guidelines.

**Table 1:** Summary of advocacy activities conducted and results on tOPV-bOPV switch in Kano State, April 2016

Stakeholder targeted	Commitment secured	Implementation
Health Commissioner (HC)	Meet with private health practitioners to solicit for cooperation on switch	The HC met with representatives of private health practitioners on 16th April 2016
	Brief media on the switch plans	The HC conducted briefing of 12 media representatives 18th April 2016
	Direct public health workers to operate outside working hours during the switch week	All public health workers were directed to be at their workplace even outside working hours during the switch
	Letter to security bodies to allow access to their facilities for the switch	Letterswere written to medical directors and commandants of Army, Airforce, Police and Immigration Service hospitals in Kano to grant access to their facilities and allow retrieval of any tOPV that may have been found
	Monitor implementation of switch plans	HC Chaired weekly review meeting on switch with the state switch committee
Director NAFDAC	Participate in pre-switch supervision of pharmaceutical and major drug dealers that stock tOPV	Provided two representatives who participated as state switch monitors that visited pharmaceutical and drug dealers that stock tOPV

**Fig. 1:**
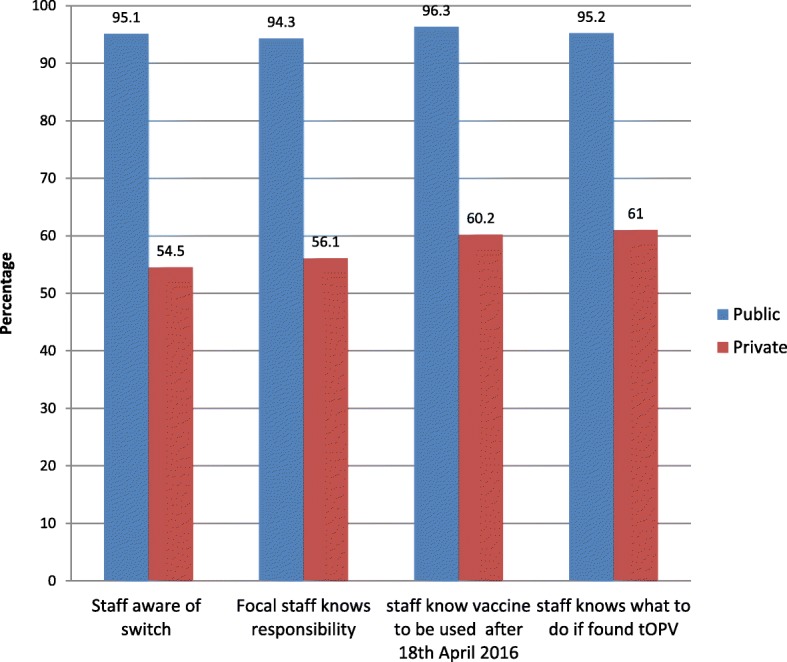
Knowledge of public and private health facility staff on switch in Kano, April 2016

A total of 670 health facilities (547 public and 123 private) that provide RI services were assessed during the pre-switch activities. Pre-switch assessment was conducted by teams of at least two persons. A total of 123 staff from government and partners participated in the exercises. Information collected during the visits, in addition to physical verification of tOPV withdrawal, included health facility staff knowledge on the switch and what to do should tOPV is found in the cold chain system. The result of the pre-switch assessment showed that 496 (91%) public health facilities had active cold chain while only 61 (49%) of private health facilities had active cold chain. Health workers were aware of the switch exercise in 520 (95.1%) of the public health facilities assessed. The health workers knew what to do should tOPV be found in any of the 521 (95.2%) of public health facilities assessed. The involvement of the private medical practitioners facilitated a hitch-free supervision and validation of private health facilities. However, there was a wide disparity between the public and private health practitioners’ knowledge on basic concepts of switch (Fig. 1).

A total of 54 cold stores were assessed during the post-switch validation process: one state, seven zonal and 44 LGA. All the 54 (100%) cold stores assessed had both active and passive cold chain. There was 100% withdrawal of tOPV from the state and the seven zonal cold stores. However, tOPVwas found in the cold chain system in 2 (4.5%) LGAs (Table 2).

**Table 2:** Switch Validation of cold stores and health facilities by Independent Monitors in Kano, April 2016

Indicators	State & Zonal Stores		LGA Stores	
	Yes		Yes	
	No	%	No	%
Has active cold chain	8	100	44	100
Has passive cold chain	8	100	44	100
tOPV in cold chain	0	0	2	4.5
tOPV out of cold chain without label “Do not use” sticker	0	0	0	0
bOPV available	8	100	44	100
IPV available	8	100	44	100

A total of 122 health facilities were validated by the independent monitors. The monitors found tOPV in the cold chain system of one (0.8%) health facility. However, there was no tOPV identified outside the cold chain without the “Do not use” sticker. Only 77.8% of the health facilities had bOPV and 79.5% had IPV(Table. 3). The findings indicate that the state had met the minimum performance required to be certified as to have successfully switched from tOPV to bOPV.

**Table 3:** Switch validation of cold chain at health facilities by independent monitors in Kano, April 2016

Indicators	Health Facilities	
	Yes	%
tOPV in Cold Chain	1	0.8
tOPV out of cold chain without label “Do not use” sticker	0	0
bOPV available	95	77.9
IPV available	97	79.5

## Discussion

Our study found that political leadership involvement in planning and implementation of the tOPV-bOPV switch in Kano State was critical to the success of the exercise. The engagement of these stakeholders by the leadership of the ministry of health facilitated their active participation in the switch. The roles played by NAFDAC and Pharmaceutical Council of Nigeria were particularly important in obtaining vital information on private pharmaceutical outfits that stock vaccines and in getting access to inspect them. Our findings re-affirmed that enlisting political support and building partnerships are key areas that need to be addressed for successful implementation of public health interventions [15]. Post switch monitoring revealed zero tOPV at the state and seven zonal satellite cold stores; this issignificant as finding tOPV in these facilities after the switch would have posedhigh riskin the distribution of tOPV to health facilities. In all, two (4.5%) LGA cold stores were found to have had tOPV that was not appropriately labeled as “Do Not Use”. More importantly, of the 122 health facilities monitored after the switch, only one (0.8%) had tOPV.

Although the tOPVwas withdrawn from the cold chain systems at the facility level, 77.8% had stock of bOPV. Also, 79.5% of the health facilities assessed had IPV. The absence of IPV in more than 20% of the health facilities after the switch is of particular concern. IPV was introduced into the RI schedule before the switch to reduce the risk of reintroduction of type 2poliovirus [6].

We also found a wide disparity in the understanding of the switch between public and private health facility staff. Only 54.5% of private health workers interviewed were aware of the switch as against 95.1% in public facilities. Also, 60.2% of workers in private facilities knew which polio vaccine to be used after the switch as against 96.3% in public facilities. This apparent low knowledge of private health workers on current polio immunization guidelines corroborates the widely held concern on the quality of care provided by private facilities in low-income countries [16].

The limitations of this study include the timing of visits to health facilities during the pre-switch supervision. As often is the case in public and private health facilities, a particular health worker is identified as the RI focal person, hence the risk of interviewing health worker with inadequate training in RI. Also, the post-switch validation involved formal RI facilities. However, there could still be private health facilities offering RI that source their vaccines elsewhere which were not necessarily known to the system.

Despite those potential limitations, the engagement of political leadership to mobilize other key stakeholders had facilitated successful implementation of the switch exercise in Kano. We will, therefore, recommend sustained advocacy to the political leadership for increased guidance in enlisting the support of critical stakeholders, private and public, in planning, implementation, monitoring and supervision of public health interventions.

## Conclusion

Nigeria switched from tOPV to bOPV in April 2016. This study demonstrated that the engagement of the political leadership to mobilize other key stakeholders facilitated successful implementation of the tOPV-bOPV switch exercise and provided opportunity to strengthen partnerships with the private health sector in Kano State.

## Abbreviations

bOPV: Bivalent oral polio vaccine
CDC: US Centers for Disease Control
cVDPV: Circulating vaccine-derived poliovirus
EOC: Polio emergency operations center
EU-SIGN: European Union Support to immunization Governance in Nigeria
IM: Incident Manager
IPV: Inactivated polio vaccine
LGA: Local government area
LQAS: Lot quality assurance sampling
MS Excel: MicrosoftExcel
NAFDAC: National Agency for Food and Drug Administration and Control
OPV: Oral polio vaccine
OPV3: Oral polio vaccine 3rd dose
PEI: Polio Eradication Initiative
PV: Polio viruses
RI: Routine immunization
SIA: Supplemental immunization activities
SPHCMB: State Primary HealthCare Development Board
tOPV: Trivalent oral polio vaccine
UNICEF: United Nations Children’s Fund
VAPP: Vaccine-associated paralytic poliomyelitis
VDPV2: Vaccine Derived Polio Virus Type 2
WHO: World Health Organization
WPV: Wild polio virus
